# Unveiling Novel Kunitz- and Waprin-Type Toxins in the *Micrurus mipartitus* Coral Snake Venom Gland: An In Silico Transcriptome Analysis

**DOI:** 10.3390/toxins16050224

**Published:** 2024-05-11

**Authors:** Mónica Saldarriaga-Córdoba, Claudia Clavero-León, Paola Rey-Suarez, Vitelbina Nuñez-Rangel, Ruben Avendaño-Herrera, Stefany Solano-González, Juan F. Alzate

**Affiliations:** 1 Escuela de Medicina Veterinaria, Universidad Bernardo O’Higgins, Santiago 8320000, Chile; 2 Centro de Investigación en Recursos Naturales y Sustentabilidad (CIRENYS), Universidad Bernardo O’Higgins, Santiago 8320000, Chile; 3 Grupo de Investigación en Toxinología, Alternativas Terapéuticas y Alimentarias, Facultad de Ciencias Farmacéuticas y Alimentarias, Universidad de Antioquia, Medellín 50010, Colombia; jessica.rey@udea.edu.co (P.R.-S.); vitelbina.nunez@udea.edu.co (V.N.-R.); 4 Escuela de Microbiología, Universidad de Antioquia, Medellín 50010, Colombia; 5 Facultad de Ciencias de la Vida & Centro de Investigación Marina Quintay (CIMARQ), Universidad Andrés Bello, Viña del Mar 2531015, Chile; ravendano@unab.cl; 6 Laboratorio de Bioinformática Aplicada, Escuela de Ciencias Biológicas, Universidad Nacional, Heredia 86-3000, Costa Rica; 7 Departamento de Microbiología y Parasitología, Facultad de Medicina, Universidad de Antioquia, Medellín 50010, Colombia; jfernando.alzate@udea.edu.co

**Keywords:** manual curations, *Micrurus mipartitus*, novel Kunitz-type inhibitor, KSPI, venom gland transcriptome

## Abstract

Kunitz-type peptide expression has been described in the venom of snakes of the Viperidae, Elapidae and Colubridae families. This work aimed to identify these peptides in the venom gland transcriptome of the coral snake *Micrurus mipartitus*. Transcriptomic analysis revealed a high diversity of venom-associated Kunitz serine protease inhibitor proteins (KSPIs). A total of eight copies of KSPIs were predicted and grouped into four distinctive types, including short KSPI, long KSPI, Kunitz–Waprin (Ku-WAP) proteins, and a multi-domain Kunitz-type protein. From these, one short KSPI showed high identity with *Micrurus tener* and *Austrelaps superbus*. The long KSPI group exhibited similarity within the *Micrurus* genus and showed homology with various elapid snakes and even with the colubrid *Pantherophis guttatus*. A third group suggested the presence of Kunitz domains in addition to a whey-acidic-protein-type four-disulfide core domain. Finally, the fourth group corresponded to a transcript copy with a putative 511 amino acid protein, formerly annotated as KSPI, which UniProt classified as SPINT1. In conclusion, this study showed the diversity of Kunitz-type proteins expressed in the venom gland transcriptome of *M. mipartitus*.

## 1. Introduction

Kunitz-type serine protease inhibitors (KSPI) are a family of protease inhibitors found in the venoms of snakes corresponding to one of the least studied toxin families. The most well-known are serine protease inhibitors. Nonetheless, a variety of additional activities have been described such as inhibitors of voltage-gated K^+^ and Ca^2+^ channels, metalloproteases, G-protein coupled receptors, enhancers of acetylcholinesterase [1], and most recently as selective antagonists to the type 2 arginine-vasopressin human receptor (hV2R) [2].

Proteome analysis of different snake venoms has demonstrated the variable abundance of these inhibitors. In the Viperidae family, KSPIs ranged from 0.3 to 28.4% of total snake venom, while in the Elapidae family, they ranged from 0.2 to 61.1% of total venom [3]. These peptides are characterized by having between 50 and 70 amino acid (aa) residues, composed of a hydrophobic core, containing a central β-sheet and a bonding pattern of three disulfide bridges in 1–6, 2–4 and 3–5 cysteine residues [4,5,6].

According to the transcript structure, they can be classified as short, long, or multi-domain KSPIs [3,7,8]. The most well-known are short KSPIs, characterized by a 24-aa-residue signal peptide that generally begins with the identical five MSSGG residues, followed by a block of five leucine residues. In the mature chain, the active site residues are located after the second cysteine residue and occupy positions 41–42, normally positively charged. After the fourth cysteine residue, there is a hydrophobic block of four residues, usually N*NN. These proteins act as competitive inhibitors and irreversibly bind to the active site of one or more proteases, and were first characterized in the study of the Bovine Pancreatic Trypsin Inhibitor (BPTI)-like protease inhibitor [9]. Short KSPIs are monomers and can also be neurotoxic, like dendrotoxins, which selectively block the Kv1.1 potassium channel, which can or cannot maintain some serine protease inhibitor activity [10,11,12].

Long KSPI transcripts have been described as having two short Kunitz-like domains and have been found in eight *Micrurus* transcriptomes [8], in other Elapids [13], and in several other Colubrids [14]. Long KSPIs have a characteristically shorter signal peptide of 20 residues that usually begin with MRREKS, and generally invariant, acidic ESPPD residues at the N-terminus. Due to high sequence homology with short KSPIs, the mature chain has putative active sites at residues 37–38 and 128–129 [8], and both short KSPIs-like domains are separated by an incomplete signal peptide sequence. Long KSPIs have only been reported via transcriptome analysis [8]; however, their functions are unknown.

Further, KSPIs have also been associated with Waprin domains and suggest a common evolution [15]. Waprin peptides consist of 50 aa residues with eight conserved cysteine residues that form four disulfide bonds. These small proteins show structural similarity to whey acidic proteins (WAP) [16] and have been associated with antibacterial activity [17]. Moreover, a Kunitz–Waprin fusion protein (Ku-WAP fusin) has been described in Viperids and Colubrids via transcriptomic analysis [4,18]. They possess the same 24-residue signal peptides, beginning with MSSGG, as short KSPIs have; however, no signal peptide sequence is found between domains, as described in long KSPIs. Waprins and Kunitz-type peptides provide an opportunity for non-conventional antimicrobial research and development [19,20,21,22].

Kunitz-type peptides were first recruited into snake venoms before the lineage split between Viperids, Elapids, and Colubrids [23,24]; therefore, they are present in all venomous snakes. Nonetheless, they are predominantly observed in Elapids, representing ~5% of their proteome on average [3,25]. Proteomic analysis of venoms of *Micrurus*, an American snake genus, commonly known as New World coral snakes, has shown that KSPIs constitute a highly prevalent protein family within *Micrurus* venoms, appearing in over 70% of the studied venoms analyzed to date [26], with a relative abundance ranging from 0.03% in the proteomic analysis of *M. lemniscatus carvalhoi* venom [27] to nearly 10.6% in *M. ruatanus* venom; the relative abundance of these inhibitors in *Micrurus* venoms is summarized in Table S1 [27,28,29,30,31,32,33,34,35,36,37,38,39]. Additionally, transcriptomic analyses of venom glands revealed the presence of Kunitz-type inhibitors in the venoms of *M. spixii* (0.65%) [40], *M. surinamensis* (0.65%) [40], *M. paraensis* (0.04%) [40], and *M. fulvius* (2.2%) [41]. In *Micruroides euryxantus*, Kunitz-type inhibitors were the most abundant protein family, accounting for 15.57% of the total transcripts. However, these toxins were not detected during proteomic analysis [42].

Proteomic analyses of Colombian *M. mipartitus* venom revealed that its composition comprises three finger toxins (3FTx) (~60%) and phospholipase A_2_ (PLA_2_) (~30%), constituting the major components, followed by L-amino acid oxidase (4.0%), P-III metalloproteinase (1.6%), Kunitz-type protease inhibitor (1.9%), serine proteinase (1.3%), and C-type lectin-like families (1.1%) [31]. Costa Rican *M. mipartitus* also contains 1.9% KSPIs [31], while these proteins were not detected in *M. mipartitus* from Ecuador [43].

In this regard, a more thorough approach to examining the venom composition of these species is needed. Transcriptomic analysis is an optimal tool for generating data platforms to search for target toxins, develop new strategies in antivenom production, and conduct overall bioprospecting studies. Based on transcriptome analyses of *M. mipartitus* venom glands from Colombia, in this work, we describe several new Kunitz-type peptides and Waprins.

## 2. Results

### 2.1. Transcriptomic Results

#### 2.1.1. General Transcriptomic Assembly Analysis and Annotation

A total of 89,375,798 reads were obtained from venom gland tissue through Illumina sequencing; 96.1% of the raw bases were above Q30 and 98.3% of reads passed the filtering process. Clean reads were assembled using Trinity v2.13.2, which obtained 84,949 contigs; Spades v3.14.1, which produced 53,604 contigs; and SOAPTRANS v1.03 kmer31 plus SOAPTRANS kmer97, which produced 218,150 contigs. ToxCodAn annotation enabled the recovery of a total of 91 curated toxin transcripts with complete coding sequences (CDS) and signal peptides from 21 toxin families. These were distributed among the four assemblies and classified according to the number of copies identified in the transcriptome, as follows: four dominant protein families including Three-Finger Toxins (3FTx-24 copies), C-type Lectins (CTL—nine copies), Snake Venom Metalloprotease (SVMP—eight copies), and Kunitz Peptides (KUN—eight copies); five secondary protein families including Lipase (LIPA—six copies), Vascular Endothelial Growth Factor (VEGF—five copies), Ficolins (five copies), Phospholipase A2 (PLA2—four copies), and Cysteine-Rich Secretory Protein (CRiSP—four copies); six minor protein families including Proteins Containing a WAP Domain (Waprins—two copies), Phosphodiesterase (PDE—two copies), Nerve Growth Factor (NGF—two copies), Phospholipase B (PLB—two copies), Vespryn (two copies), and Hyaluronidase (HYAL—two copies); and six rare protein families including Snake Venom Serine Protease (SVSP—one copy), Acetylcholinesterase (AChE—one copy), L-amino Acid Oxidase (LAO—one copy), Complement C3 (VCO3—one copy), Coagulation Factor Xa inhibitory (CFX—one copy), and Natriuretic Peptides (NP—one copy).

#### 2.1.2. Transcriptomic Analysis Revealed Novel Sequences of Kunitz-Type Inhibitors (KSPI)

Transcriptomic analysis of the venom gland of the coral snake *M. mipartitus* showed highly diversified venom proteins of the Kunitz-type serine protease inhibitors (Kunitz/BPTI inhibitors or KSPIs). A total of eight copies of KSPIs (*M.m*-C482594, *M.m*-NODE-3517, *M.m*-NODE-13319, *M.m*-NODE-19949, *M.m*-NODE-19547, *M.m*-NODE-21634, *M.m*-TRINITY-DN 744, and *M.m*-scaffold14755) were grouped in four distinctive classes, annotated by ToxCodAn and hand-curated. These included short Kunitz, long Kunitz, Ku-WAP and Kunitz-type proteins with two or more domains, referred to as multi-domain from now on. All these transcripts present signal-peptide (Likelihood 0.9997) and glycosylation sites, indicating that their protein product is secreted.

From Trinity’s assembly, the relative abundance of the transcripts for this protein family were quite high, representing 6.4% of the total transcripts per million (TPM) of the toxin transcripts. From Spades assembly, the TPM count was 12.0%; whereas or while, with SOAPTRANS k-mer size of 31, the TPM count was 3.1%. This relative expression level was calculated adding up the TMP values of each individual KSPI ToxCodAn annotated transcript, identified by each individual assembler, among the total toxin annotated transcripts.

From these four Kunitz groups, the most expressed Kunitz transcripts corresponded to the short Kunitz, accounting for 98% of the total toxin expression within the family. The three assemblers identified the short Kunitz transcripts as the most expressed group. Ku-WAP fusin transcripts, (0.65%) were recovered only from Trinity and Spades assemblies, whereas long Kunitz (0.4%) were recovered from all three assemblies. Finally, multi-domain Kunitz transcripts close to 0% were found only by SOAPTRANS v1.03 k-mer 31.

**Figure 1:**
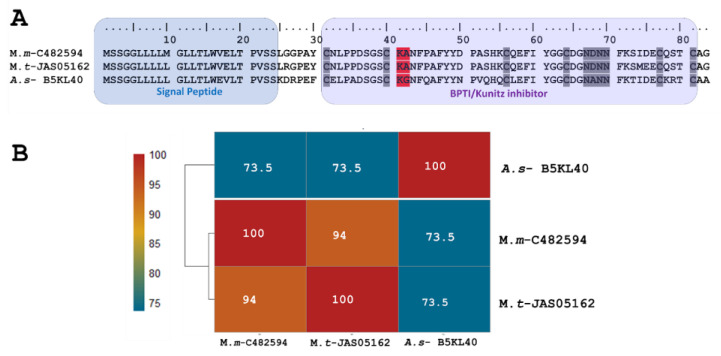
Analysis of the putative short KSPIs identified in the *M. mipartitus* transcriptome. (**A**) Deduced amino acid sequence alignment of short Kunitz *M.m*-C482594 (accession number: PP439990) found in the *M. mipartitus* transcriptome with related sequences from public databases (*Micrurus tener (M.t)* and *Austrelaps superbus (A.s))*. Colored backgrounds indicate the signal peptide and Kunitz domains. Active site residues are indicated in red. Cysteines and the block of hydrophilic aa residues are indicated in gray. (**B**) Pairwise sequence similarity is highlighted according to the percent identity shown in the left-hand column.

The short KSPI copy *M.m*-C482594 (PP439990) identified in *M. mipartitus* showed an identity of 94% and 73.5% with *M. tener* (A0A194ARF4-UniProtKB) and *Austrelaps superbus* (B5KL40-UniProtKB), respectively. This short KSPI predicted in *M. mipartitus* has a typical 24-aa-residue signal peptide beginning with MSSGGL and 59 aa residues in the mature chain (Figure 1). In *M. mipartitus*, K aa and A aa were observed at the active site position (41–42) and presented a block of hydrophilic aa residues NDNN at positions 67–70.

**Figure 2:**
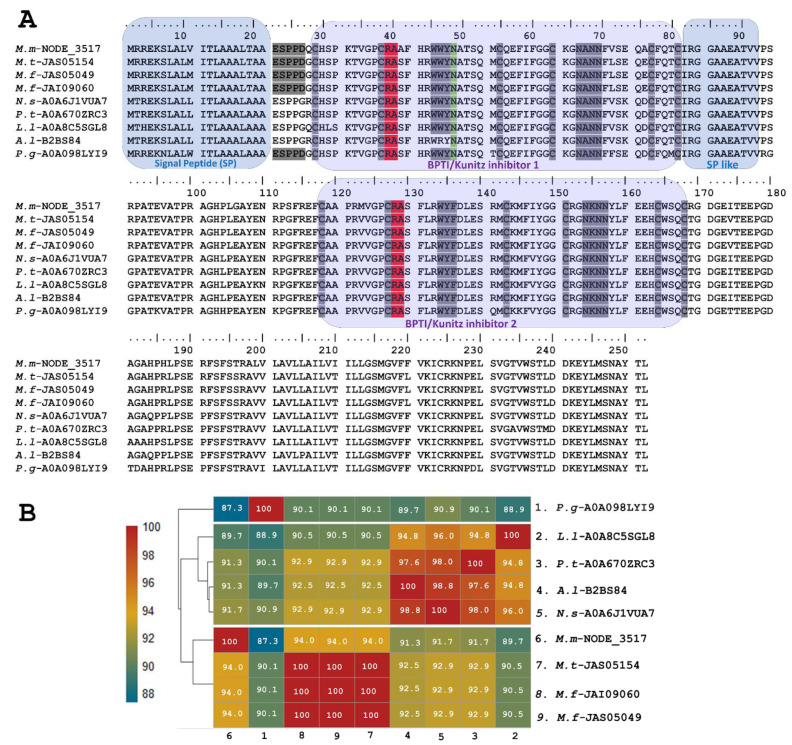
Analysis of the putative long KSPIs identified in the *M. mipartitus* transcriptome. (**A**) Deduced amino acid sequence alignment of long Kunitz *M.m*-NODE-3517 (accession number PP439991) found in *M. mipartitus* transcriptome with related sequences from public databases (M. tener (*M.t*), *M. fulvius* (*M.f*), *Notechis scutatus* (*N.s*), *Pseudonaja textilis* (*P.t*), *Austrelaps labialis* (*A.l*)*, Laticauda laticadus* (*L.l*), and *Pantherophis guttatus* (*P.g*)). The signal peptide and double Kunitz domains are highlighted by colored backgrounds. Active site residues are indicated in red, and N-linked glycosylation in position 46 is indicated in green. Cysteines and the block of hydrophilic aa residues are indicated in gray. (**B**) Pairwise sequence similarity is highlighted according to percent identity shown in the left-hand column.

The sequence of putative long KSPIs identified in *M. mipartitus* (*M.m*-NODE_3517; PP439991) was compared to counterparts and showed a higher percentage of identity (94%) with other *Micrurus* species such as *M. tener* (JAS05154-UniProtKB) and *M. fulvius* (JAS05049, JAI09060-UniProtKB) (Figure 2). Moreover, the study revealed that these toxins also exhibit homology with those derived from other elapids, including genera such as *Notechis, Pseudonaja*, and *Austrelaps*, as well as with the colubrid species *Pantherophis guttatus*. These long KSPIs in the *Micrurus* genus are characterized by a 20-aa-residue signal peptide that initiates with the sequence MRREKS, which contrasts with the MTREKS initial sequence observed in other genera within the Elapidae family. Long KSPIs from the *Micrurus* genus and from *P. guttatus* (Colubridae family) have a well-conserved, acidic N-terminus (ESPPD); however, in other Elapidae genera, these acidic N-terminal residues are ESPPG. The potential BPTI/Kunitz inhibitor 1 domain position ranged from residues 27 to 77 with R and A aa residues in active site positions (37–38). In addition, a strongly aromatic WWY sequence (residues 43–45), asparagine N-linked glycosylation in position 46, and four hydrophilic residues, NANN, found at positions 63–66 were observed.

Towards the C-terminal, the protein sequence of the toxin continues with ~11 aa residues that are mainly aliphatic, mimicking a signal peptide. The position of the BPTI/Kunitz inhibitor 2 domain ranged from residues 118 to 168 with R and A aa residues located at active site positions 128 and 129. Positions 134–136 show another aromatic triplet, WYF. The second domain also has a block of hydrophilic residues (positions 154–157: NKNN).

**Figure 3:**
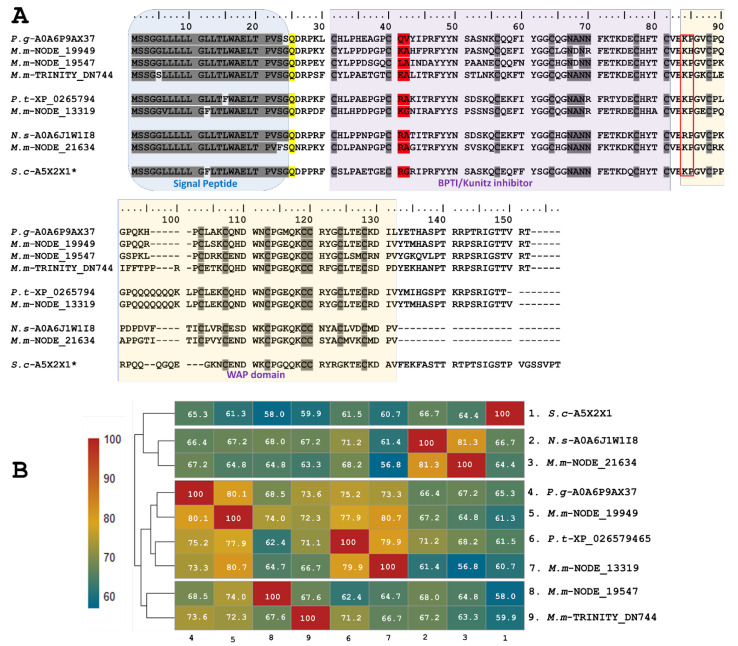
Analysis of the putative Ku-WAP fusin sequences identified in *M. mipartitus* transcriptome (*M.m*-NODE-13319, PP439993; *M.m*-NODE-19949, PP439992; *M.m*-NODE-19547, PP439994; *M.m*-NODE-21634, PP439996; and *M.m*-TRINITY-DN744, PP439995). (**A**) Deduced amino acid sequence alignment of the Ku-WAP fusin sequences found in *M. mipartitus* with related sequences from public databases (*Pantherophis guttatus* (*P.g*), *Pseudonaja textilis* (*P.t*), *Sistrurus catenatus edwardsii* (*S.c*), and *Notechis scutatus* (*N.s*)). * Ku-WAP fusin identified in viperids with loss of second cystein residue of the WAP domain. The different domains are highlighted by colored backgrounds. The modified residue in position 25 (Q- Pyrrolidone carboxylic acid) is depicted in yellow, the active site at positions 41-42 is depicted in red, and the red box indicates the potential cleavage site between Kunitz and WAP domains in Ku-WAP-fusin. Cysteines and the block of hydrophilic aa residues are indicated in gray. (**B**) Pairwise sequence similarity is highlighted according to percent identity shown in the left-hand column.

Five identified transcripts, *M.m*-NODE-13319 (PP439993), *M.m*-NODE-19949 (PP439992), *M.m*-NODE-19547 (PP439994), *M.m*-NODE-21634 (PP439996), and *M.m*-TRINITY-DN744 (PP439995), enabled the inference of larger proteins containing Kunitz domains in addition to a WAP (whey-acidic-protein-type four-disulfide core) domain (Figure 3). Three of these (*M.m*-NODE-19949, M.m-NODE-19547, and *M.m*-TRINITY-DN 744) exhibited similarity to Ku-WAP-fusin toxins (A0A6P9AX37-UniProtKB) described in the colubrid *P. guttatus*. Furthermore, the copy *M.m*-NODE-13319 showed correspondence with *P. textilis* (XP_026579465-GenBank) and the copy *M.m*-NODE-21634 with *N. scutatus* (A0A6J1W1I8-UniProtKB).

The predicted translated transcripts identified in *M. mipartitus* show an identity of ~60% to Ku-WAP-fusin toxins, initially described in vipers, specifically in *Sistrurus catenatus edwardsii* (A5X2X1-UniProtKB). Alignment of these sequences (Figure 3) presents a non-conserved signal peptide of 24 aa residues, a modified residue in position 25 (Q- Pyrrolidone carboxylic acid), and a block of hydrophilic residues (positions 67–70, N*N*) in which the asterisk may be A/D and N/R, for the first and second asterisk, respectively.

The active site at positions 41–42 varies among different copies; aa residues RA, KG and KA may determine a reactive bond for trypsin or plasmin, while LA residues might indicate a reactive bond for chymotrypsin. On the other hand, EA residues may exhibit non-inhibitory KSPI-like behavior upon substitution with a negatively charged residue. The potential cleavage site (KP) between Kunitz and WAP in Ku-WAP toxins is conserved in all *M. mipartitus* copies. Every copy of *M. mipartitus* displayed a characteristic four-disulfide core typical of whey acidic protein types retaining the second Cys residue of the WAP domain.

#### 2.1.3. Single and Double Waprin Domains in the *M. mipartitus* Transcriptome

We also found putative Waprin-like toxins in the transcriptome of *M. mipartitus* venom glands, in which WAP domains occur solely without the Kunitz domain. WAP toxin family expression represents up to 2% of the total annotated toxin transcripts. Two copies of Waprin were identified, one with a double Waprin domain (*M.m*-NODE_13492; PP439998), equivalent to 98% of the total family expression, and the other copy with a single Waprin domain (*M.m*-C493867; PP439999), which represented 1.9% of total WAP expression.

**Figure 4:**
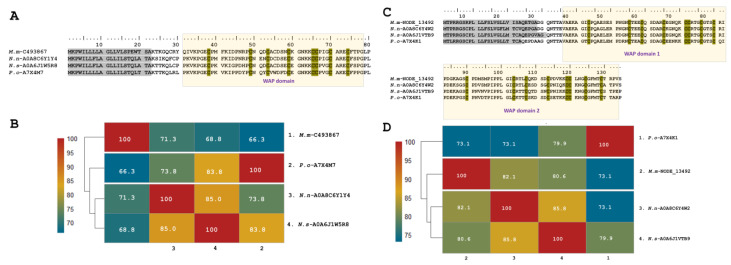
Analysis of the putative Waprin sequences identified in the *M. mipartitus* transcriptome. Deduced amino acid sequence alignment of (**A**) single (*M.m*-C493867; PP439999) and (**C**) double (*Mm*-NODE_13492; PP439998) Waprin domains with related sequences published in the database. Signal peptides are indicated in gray, and the WAP domain is shown with a yellow background. Cysteines are indicated in gray. Pairwise sequence similarity is highlighted according to percent identity shown in the left-hand column. (**B**) Single Waprin domain and (**D**) double Waprin domain. *Micrurus mipartitus* (*M.m*), *Naja naja* (*N.n*), *Notechis scutatus* (*N.s*) and *Philodryas olfersii* (*P.o*).

Single Waprins in *M. mipartitus* showed an identity of 71.3% with *Naja naja* (A0A8C6Y1Y4-UniProtKB), 68.8% with *Notechis scutatus* (A0A6J1W5R8-UniProtKB) and 66.3% with *Philodryas olfersii* (Waprin-Phi3, A7X4M7-UniProtKB). Single Waprins present a signal peptide of 22-residues and consist of 58-residues in mature chains (Figure 4A,B). The WAP domain (31–78 positions) comprises eight cysteine residues involved in four disulfide bonds.

Waprin transcripts from *M. mipartitus* encoding two WAP domains showed a similarity of 82.1% with *Naja naja* (A0A8C6Y4W2-UniProtKB), 80.6% with *N. scutatus* (A0A6J1VTB9-UniProtKB), and 73.1% with *P. olfersii* (Waprin-Phi1, A7X4K1-UniProtKB) (Figure 4C,D). The signal peptide length showed differences among these sequences, corresponding to 28 residues in *M. mipartitus* and *N. naja*, 30 residues in *N. scutatus*, and 23 residues in *P. olfersii*.

#### 2.1.4. Multidomain Putative Annotated as Kunitz by ToxCodAn Shows Similarity to SPINT1 Human Gene

**Figure 5:**
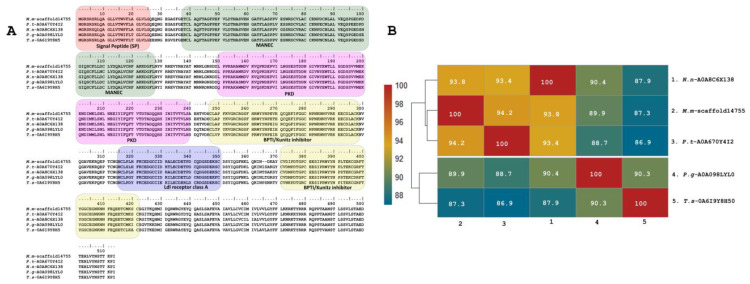
Deduced amino acid sequence alignment of multidomain putative protein (*M.m*-scaffold14755, PP439997) annotated as Kunitz identified in the *M. mipartitus* transcriptome with related sequences published in the database (**A**). Pairwise sequence similarity is highlighted according to percent identity shown in the left-hand column (**B**). The signal peptide is indicated in red. Active sites for each Kunitz domain are indicated as RG and EE residues. *Micrurus mipartitus* (*M.m*), *Pseudonaja textilis* (*P.t*), *Naja naja* (*N.n*), *Pantherophis guttatus* (*P.g*), and *Thamnophis sirtalis* (*T.s*).

In addition to the described peptides, a predicted copy of 511 residues was annotated as Kunitz (with a 25-residue signal peptide) (M.m-scaffold14755, PP439997) in the transcriptome of *M. mipartitus.* This putative protein has a 51.6% identity to the SPINT1 human gene (serine peptidase inhibitor, Kunitz type 1 protein), a hepatocyte growth factor activator inhibitor 1 (HAI-1) multi-domain protein. The *M. mipartitus* variant of this protein is likewise a membrane-bound multidomain protein containing an extracellular region that consists of a MANEC (91 residues, motif at N terminus with eight cysteines), an internal PKD-like domain (90 residues), Kunitz BPTI domain 1 (52 residues), a low-density lipoprotein receptor class A (LDLRA) domain (36 residues), and Kunitz domain 2 (51 residues) followed by a C-terminal single-span transmembrane region (Figure 5A,B). This sequence showed similarity to *Pseudonaja textilis* (94.2%, A0A670Y4I2-UniProtKB), *Naja naja* (93.8%, A0A8C6X138-UniProtKB), *Pantherophis guttatus* (89.9%, A0A098LYL0-UniProtKB) and *Thamnophis sirtalis* (87.3%, A0A6I9Y8H5-UniProtKB).

## 3. Discussion

Our findings support the existing literature on the distribution of Kunitz-type toxins in several *Micrurus* species [44,45]. The diversity of these toxins underscores the adaptability of snakes to different ecological niches. The inhibitory action of Kunitz-type toxins on specific serine proteases, notably those involved in blood clotting and fibrinolysis, aligns with the prevailing hypothesis that these toxins play a pivotal role in prey immobilization [46]. These snake venom peptides greatly vary in their targeting specificity. The function of inhibitors of different serine proteases seems to be related to modifications in prey homeostasis, especially in the blood coagulation cascade and anticoagulant effect, a critical component of the envenomation strategy adopted by snakes [47].

In this work, eight copies of KSPIs were identified in *M. mipartitus*. The relative expression levels of transcripts for this protein family identified by the three implemented assembler types (3.1%, 6.4% and 12%) were higher than for other transcriptomes described in other *Micrurus* species such as *M. surinamenesis* (0.006%), *M. spixi* (0.007%), and *M. paraensis* (0.0003%). However, they were comparable with other *Micrurus* species like *M. lemniscatus*, where the expression of this toxin family is 7.1% [8].

The most expressed transcript of the variants corresponded to a short Kunitz protease. This finding agrees with a serine protease inhibitor and possibly a trypsin inhibitor, which was manually inferred from sequence similarity to B5KL40-UniProtKB (Figure 1). It is known that the amino acid residues present on the binding loop of Kunitz-type protease inhibitors determine their specificity and potency for different serine proteases. Residues P, L, and Y, for example, confer chymotrypsin specificity; residues K and R tend to confer inhibition of trypsin and trypsin-like enzymes, while A and S lead to the inhibition of elastase-like enzymes [48,49]. Short Kunitz predicted in this work showed KA residues in active sites (positions 41–42), like those presented in a Kunitz-type peptide transcript in *Micrurus tener* [50] and a Kunitz-type peptide isolated from *Vipera ammodytes* (P00991-UniProtKB), a KA serine protease inhibitor that principally inhibited trypsin (Ki = 0.34 nM). Similarly, it also inhibits alpha-chymotrypsin (Ki = 270 nM), plasmin, plasma, and pancreatic kallikrein [51].

Several Kunitz-type serine protease inhibitors (KSPIs) that act on trypsin have been isolated from elapids. One KSPI isolated from the venom of *Bungarus fasciatus* inhibited trypsin, chymotrypsin, and elastase [52], while another from *Oxyuranus scutellatus* showed inhibition against trypsin, elastase, and kallikrein. However, the latter, presenting an R residue, was more potent against plasma kallikrein [48]. Several KSPIs with trypsin inhibition activity have also been reported in *Naja* sp., *Ophiophagus hannah*, *Pseudonaja textilis textiles*, and *Pseudechis australis* [53]. Only one *Micrurus* Kunitz-type peptide has been isolated and characterized, tenerplasmininin-1 isolated from *Micrurus tener*, which showed a molecular mass of 6542 Da, and, as with others, inhibited the plasmin-mediated degradation of fibrinogen chains (plasmin inhibition), trypsin, and elastase [53,54].

On the other hand, Kunitz-type peptides with neurotoxic action are mainly found in elapid snakes and dendrotoxins from mambas, from which they were initially identified [2]. The action mechanisms of these facilitate the release of acetylcholine, a spastic paralysis inducer [1]. Therefore, it would be beneficial to determine if the Kunitz-type peptide transcripts found in this work have neurotoxic activity and if they possibly contribute to the neurotoxicity caused by *M. mipartitus* venom, and/or further other neurotoxic toxins such as Three Finger toxins or Phospholipases A_2_ [31].

In contrast, none of the putative KSPI sequences identified in this study matched MitTx, a heterodimeric protein complex initially discovered in the venom of *M. tener*. This complex comprises a Kunitz-like subunit (Mit-α) that binds non-covalently to a catalytically inactive PLA2 homolog (MitTx-β) [55]. It has also been found in the venom of the long-tailed monadal Central and North American coral snakes *M. mosquitensis*, *M. nigrocinctus* [37], and *M. browni* [34], the long-tailed monadal South American coral snake *M. dumerilii* [36,47], and, most recently, *M. frontalis*. This represents the first species of the short-tailed triad coral snake complex from South America [27]. The involvement of the heterodimeric toxin in the ecological–evolutionary context and envenoming strategies of the coral snake is yet to be fully understood.

The long Kunitz *M. mipartitus* transcript found in this analysis showed very low expression levels (0.0064% of the total annotated toxin transcripts), similar to levels detected in *M*. *surinamensis* (0.006%), *M. s. spixii* (0.007%), and *M. paraensis* (0.0003%), while total expression of the KSPIs family was approximately 0.65%, 1.1%, and 0.4%, respectively [8]. In contrast, in the transcriptome of *M. mipartitus*, higher percentages of putatively identified as Kunitz-type peptides were obtained, which questions the role of long Kunitz proteins in prey envenomation. To our knowledge, long Kunitz proteins have only been described in elapids and several other colubrids; however, the function of these proteins has yet to be described.

Five transcripts encoding larger proteins containing KSPI domains fused to a WAP domain (Ku-WAP-fusin) were identified. Together, these Ku-WAP-fusin transcripts represented 0.65% of the total Kunitz expression found in this study. This constitutes the first report of this type of transcript in *Micrurus* venoms. However, in old-world coral snakes, such as *Calliophis intestinalis*, Ku-WAP-fusin was found in percentages up to 2% [56]. It is still unknown if the potential cleavage site (KP) described between the Kunitz and WAP domains in Ku-WAP toxins is processed post-translationally or if these proteins act as a complex. The discovery of these types of proteins in old-world coral snakes like *C. intestinalis* and *M. mipartitus*, an ancestral species within the *Micrurus* genus [57], may suggest the basal diversification of this protein family. In contrast, heterodimers composed of Kun/PLA_2_ have been described in the venoms of evolutionarily more recent snakes, such as *M. dumerilii*, *M. nigrocinctus*, and *M. mosquitensis* [57,58].

Similarly, Waprins have been identified in several elapid snake venoms. Nawaprin was the first WAP domain protein identified in snake venom and was isolated from *N. nigricollis* [59]. Similarly, Omwaprin was isolated from *Oxyuranus microlepidotus,* a protein of 50 aa residues and eight cysteines. Further, Omwaprin revealed activity against some Gram-positive bacteria, but not against Gram-negative strains [17]. Waprin-like sequences are present in *M. corallinus*, *M. lemniscatus carvalhoi*, *M. lemniscatus lemniscatus, M. paraensis*, *M. spixii spixii*, and *M. surinamensis* [8]. The predicted mature chain of Waprins identified in this work presented 58 aa residues and the same amount of cysteine residues, similar to *Naja naja* and *Notechis scutatus* and the colubrid *Philodryas olfersii.* Double-WAP domain transcripts had been reported only in the venom of the South American arboreal colubrid *Phylodryas olfersii* [24]. Signal peptide sequences in single and double WAP transcripts differ considerably between short and long KSPIs, challenging the idea of a common ancestry, as mentioned in Jackson et al. [13]. To isolate and characterize the putative Waprins found in this work, it would be helpful to determine their antimicrobial potential and possible evolutionary connections between various snake species.

Snake venom contains a variety of proteins with diverse functions, including toxins that may mimic or share similarities with human proteins. Hepatocyte Growth Factor Activator Inhibitor 1 (HAI-1) is one of them. HAI-1 is a multidomain Kunitz-protease inhibitor that plays an important role in regulating proteolytic and morphogenic processes [60]. In the context of snake venom, these toxins likely evolved to target host hemostasis and immune responses, aiding in prey immobilization or defense. Furthermore, like HAI-1, it could participate by inhibiting different venom enzymes and thus prevent self-degradation or activation, and, similarly to HAI-1, the MANEC and Kunitz 2 domains could contribute to the inhibitory effect [61]. The presence of HAI-1-like toxins in snake venoms underscores the complexity of venom composition and suggests potential pharmacological applications, such as anti-cancer properties through the inhibition of cancer cell migration [62,63].

Our study contributes to the broader understanding of venom evolution by highlighting the significant variability in Kunitz-type and WAP toxins among different snake species. This diversity suggests a complex interplay between evolutionary pressures, ecological niches, and prey preferences [64,65]. The identification of KSPI toxins with specific activities over different serine proteases or more possible therapeutic targets opens new avenues for exploring their therapeutic potential. Proteases and peptidases regulate a broad variety of processes present in all living organisms, such as viruses’ life cycle development, coordinating entry, maturation, assembly and bursting out of host cells [66]. In nematodes, proteases regulate cyst development, a relevant stage in zoonosis [67]. Additionally, proteases participate in immune response and microbial infection in plants, insects, and fungi [68,69] and a plethora of bleeding and inflammation-related processes in animals, as well as in the nervous system, as voltage-gated ion channels are involved in critical processes related to nerve cells [70], making them versatile tools in many fields. Recently, several KSPI peptides from green mamba venom have been tested for effects on cyst development in polycystic kidney diseases via the inhibition of vasopressin type 2 receptor pathways [2,70].

Further investigations into the structural features of Kunitz-type toxins are warranted. Understanding the structure–function relationships can provide insights into their mode of action and guide the development of targeted interventions [71,72]. While acknowledging the limitations of our study, such as the need for more extensive sampling across diverse snake taxa, we feel that this analysis offers diverse strategies for future research. Investigating the transcriptomic and proteomic profiles of snake venom has revealed novel insights into the complexity of Kunitz-type toxins. Additionally, it might reveal potential differences at regulatory levels, such as differences between the transcriptome and proteins found in venom glands. It is crucial to identify key peptides that might not be visible by studying only one information level; therefore, a multidimensional study is key for a comprehensive understanding of the variety, structure, function, and effect of snake venom peptides.

## 4. Conclusions

The main findings are that 21 protein families constitute *M. micrurus* toxins. The four dominant protein families include Three-Finger Toxins, C-type Lectins, Snake Venom Metalloprotease, and Kunitz Peptides. Secondary protein families include Lipases, Vascular Endothelial Growth Factor, Ficolins, Phospholipase A2 and Cysteine-Rich Secretory Protein, in addition to six minor protein families. Transcriptome analysis identified diverse sequences of Kunitz-type inhibitors, including Waprin-like toxins, and predicted secreted and membrane-bound peptides.

Our study provides valuable knowledge of Kunitz-type toxins in *Micrurus* snake venom. By elucidating their functional significance, evolutionary implications, and potential applications, we hope to stimulate further research in this intriguing field. Additionally, we demonstrated that implementing a hybrid assembler approach increases the probability of identifying low-abundance copies, as we recovered new ones from the transcriptomic analysis.

## 5. Materials and Methods

### 5.1. Biological Samples and RNA Extraction from Venom Gland Tissue

The *M. mipartitus* venom gland tissue used in this study was provided by the serpentarium tissue bank of the Toxinology, Therapeutic and Food Alternatives research group at the University of Antioquia (Colombia). Venom gland tissue had been isolated from an 88.2 cm adult *M. mipartitus* female collected in the region of Antioquia, Colombia. Venom gland tissue was meticulously excised and preserved in RNAlater^®^ for subsequent RNA isolation, immediately after the snake’s natural death. The use of this biological material has the approval of the Ministry of Environment and Sustainable Development of Colombia, under the contract for access to genetic resources and their derivative products, N°370 and 126 (addendum # 14).

The biological sample was transported in liquid nitrogen to the Centro Nacional de Secuenciación Genómica of the University of Antioquia. Upon arrival, the tissue was immediately suspended in Trizol^TM^ Reagent (Invitrogen; Waltham, MA, USA) and processed following the manufacturer’s instructions. The RNA concentration and quality was measured using capillary electrophoresis with an Agilent Bioanalyzer 2100 (Agilent, Santa Clara, CA, USA).

### 5.2. Transcriptome Sequencing and Quality Analysis

A 150 bp paired-end cDNA library was prepared using TruSeq Stranded mRNA (Illumina, San Diego, CA, USA). Sequencing was performed using an Illumina Novaseq 6000. Reads were trimmed for adapters and quality using CUTADAPT v3.5 software [73]. The quality trimming filter was set at Q30, and after processing, reads with lengths below 70 base pairs were discarded.

### 5.3. De Novo Assembly and Quality Control Analysis

The assembly of the snake venom gland transcriptome was successfully achieved employing several assemblers [74,75,76]. Three assemblers were used, and the combined results were subjected to redundancy filtering for a final data set. The de novo assemblers were Trinity v2.13.2 [77] with default parameters, sPAdes v3.14.1 [78] (k-mer, k = 31) and SOAPdenovo-trans v1.03 [79] with k-mer 31 and 97, selected to generate a diverse array of assembled transcripts. High-quality reads (≤Q30) were assembled into contigs using these three approaches. Transcript abundance was measured using Kallisto 0.44.0, TPMsfbvags [80] by estimating the read count for all transcripts as transcripts per million (TPM). To calculate the relative expression level for each assembler individual toxin, annotated TPM transcript values were manually categorized and summed up, according to the toxin family’s annotation made by ToxCodAn. Then, calculated values were expressed as a percentage of the total annotated transcript. Transcriptome statistics were obtained using a custom Python script written by the Centro Nacional de Secuenciación Genómica of the University of Antioquia.

### 5.4. Functional Annotation of Transcriptome

Functional annotation of non-toxins, putative toxins and toxin transcripts was performed with the ToxCodAn v1.0 package, available at https://github.com/pedronachtigall/ToxCodAn (package downloaded and installed on 5 May 2023) [81]. Briefly, the ToxCodAn pipeline comprises a series of steps that allow toxin annotation, by contrasting assembled sequences with a curated toxin database (toxinDB), to accurately assign the CDS function. Three main FASTA files were created from assembled contigs: (1) non-toxins, obtained by having no hits against the toxinDB; (2) putative toxins (coverage < 80% against toxinDB); and (3) toxins (coverage > 80%). These three sets of sequences passed through a signal peptide prediction step using SignalP v4.1 to filter toxins without signal peptides and a redundancy removal step involving clustering the toxins and putative toxin sequences with 100% identity in size and sequence, using an in-house Python script.

### 5.5. Manual Sequence Curation

Toxin and putative toxin sequences were blasted against the GenBank https://www.ncbi.nlm.nih.gov/genbank/ (accessed on 11 June 2023) and Uniprot databases https://www.uniprot.org/ (accessed on 11 June 2023) and aligned with the highest hit. Length; signal peptide sequences in the SignalP v6.0 server, available at https://services.healthtech.dtu.dk/services/SignalP-6.0/ (accessed on 11 June 2023) [82]; conserved domain identification, available at https://www.ncbi.nlm.nih.gov/Structure/cdd/cdd.shtml (accessed on 11 June 2023); and the number of disulfide bonds were validated manually for each sequence according to each toxin family. All chimera sequences were removed. Heat maps showing identity percentage were made with RStudio 24.04.0 [83]

## Data Availability

Annotated nucleotide sequences were deposited in the GenBank database under accession numbers PP439990–PP439999.

## Supplementary Materials

The following supporting information can be downloaded at: https://www.mdpi.com/article/10.3390/toxins16050224/s1, Table S1: Quantitative summary of estimates of Kunitz-type serine protease inhibitor abundances (% of total protein content) in venom from some *Micrurus* species analyzed using the proteomics strategy.
