# Supplementary material for: Unveiling Novel Kunitz- and Waprin-Type Toxins in the Micrurus mipartitus Coral Snake Venom Gland: An In Silico Transcriptome Analysis
Source: Toxins (Basel). 2024 May 11;16(5):224. doi: 10.3390/toxins16050224 (PMC11126030; doi:10.3390/toxins16050224)
Supplement: Supplementary file 1 [file toxins-16-00224-s001.zip › toxins-2926880-supplementary.pdf]

Table S1. Quantitative summary of estimates of Kunitz-type serine protease inhibitor abundances (% of total protein content) in venom from some *Micrurus* species analyzed by the proteomics strategy.

***Micrurus* species**	**Relative abundance in percentage**	**Reference**
M. lemniscatus carvalhoi	0.03	Sanz et al., 2019 [27]
M. clarkii	0.9	Lomonte et al., 2016 [28]
M. yatesi	0.9	Mena et al., 2022 [29]
M. frontalis	1.0	Sanz et al., 2019 [27]
M. spixi spixi	1.1	Sanz et al., 2019 [27]
M. tschudii tschudii	1.6	Sanz et al., 2016 [30]
M. multifasciatus	1.9	Rey-Suarez et al., 2011 [31]
M. mipartitus	1.9	Rey-Suarez et al., 2011 [31]
M. altirostris	2.1	Corrêa-Netto et al., 2011 [32]
M. helleri (M. lemnisctaus helleri)	2.2	Rodriguez-Vargas et al., 2023 [33]
M. browni browni	2.7	Bérnard-Valle et al., 2020 [34]
M. sangilensis	2.9	Rodriguez-Vargas et al., 2023 [33]
M. pyrrhocryptus	5.0	Olamendi-Portugal et al., 2018 [35]
M. medemi	5.3	Rodriguez-Vargas et al., 2023 [33]
M. helleri (M. lemnisctaus helleri)	5.9	Sanz et al., 2019 [27]
M. dumerilii	9.0	Rey-Suarez et a., 2016 [36]
M. ibiboboca	9.4	Sanz et al., 2019 [27]
M. mosquitensis	9.8	Fernandez et al., 2015 [37]
M. ruatanus	10.6	Lippa et al., 2019 [38]

*M. nigrocinctus**	ND	Fernandez et al., 2011 [39], Fernandez et al., 2015 [37]

* In the proteomic analysis of *M. nigrocinctus* venom, Kunitz was not identified. However, in the study by Fernandez et al., 2015, a subsequent search was conducted in the database, revealing peptides that matched the Mit-Tx complex.
